# Altered muscle membrane potential and redox status differentiates two subgroups of patients with chronic fatigue syndrome

**DOI:** 10.1186/s12967-020-02341-9

**Published:** 2020-04-19

**Authors:** Yves Jammes, Nabil Adjriou, Nathalie Kipson, Christine Criado, Caroline Charpin, Stanislas Rebaudet, Chloé Stavris, Régis Guieu, Emmanuel Fenouillet, Frédérique Retornaz

**Affiliations:** 1 UMR 1263 C2VN INRA INSERM, Faculty of Medicine, Aix Marseille University, Marseille, France; 2 Department of Internal Medicine, European Hospital, Marseille, France; 3 Institut National des Sciences Biologiques, CNRS, Paris, France

**Keywords:** Muscle excitability, Oxidative stress, Potassium outflow, Myalgic encephalomyelitis/chronic fatigue syndrome

## Abstract

**Background:**

In myalgic encephalomyelitis/chronic fatigue syndrome (ME/CFS), altered membrane excitability often occurs in exercising muscles demonstrating muscle dysfunction regardless of any psychiatric disorder. Increased oxidative stress is also present in many ME/CFS patients and could affect the membrane excitability of resting muscles.

**Methods:**

Seventy-two patients were examined at rest, during an incremental cycling exercise and during a 10-min post-exercise recovery period. All patients had at least four criteria leading to a diagnosis of ME/CFS. To explore muscle membrane excitability, M-waves were recorded during exercise (rectus femoris (RF) muscle) and at rest (flexor digitorum longus (FDL) muscle). Two plasma markers of oxidative stress (thiobarbituric acid reactive substance (TBARS) and oxidation–reduction potential (ORP)) were measured. Plasma potassium (K^+^) concentration was also measured at rest and at the end of exercise to explore K^+^ outflow.

**Results:**

Thirty-nine patients had marked M-wave alterations in both the RF and FDL muscles during and after exercise while the resting values of plasma TBARS and ORP were increased and exercise-induced K^+^ outflow was decreased. In contrast, 33 other patients with a diagnosis of ME/CFS had no M-wave alterations and had lower baseline levels of TBARS and ORP. M-wave changes were inversely proportional to TBARS and ORP levels.

**Conclusions:**

Resting muscles of ME/CFS patients have altered muscle membrane excitability. However, our data reveal heterogeneity in some major biomarkers in ME/CFS patients. Measurement of ORP may help to improve the diagnosis of ME/CFS.

*Trial registration* Ethics Committee “Ouest II” of Angers (May 17, 2019) RCB ID: number 2019-A00611-56

## Background

Adult patients with chronic fatigue may be suffering from chronic fatigue syndrome, also known as myalgic encephalomyelitis/chronic fatigue syndrome (ME/CFS). ME/CFS often occurs in the absence of any disease that could be responsible for body fatigue and is characterized by intense fatigue which is worsened by physical/mental activity and is often associated with post-exertional malaise (PEM) [1, 2]. Current definitions of ME/CFS do not use biological markers.

Several body systems including the muscular and nervous systems are affected in ME/CFS [3–5]. Potential causes of muscle dysfunction in ME/CFS patients may include oxidative stress with reduced heat-shock protein production. Experimental evidence indicates that exercising skeletal muscles are often affected in ME/CFS because marked alterations in myopotential occur in response to direct muscle stimulation (M-wave) [5–11]. M-wave alterations show impaired muscle membrane excitability and begin early in the exercising muscles before culminating during the 10-min recovery period [6–8]. In contrast, M-wave alterations are absent in healthy subjects in whom the myopotential amplitude often increases with the incremental pedalling force [12, 13]. No data are available on altered excitability in the resting muscles of ME/CFS patients.

Fulle et al. [7] observed a dysregulation of the Na^+^/K^+^ ATPase pump in ME/CFS patients and suggested that this dysregulation could result from increased fluidity of the sarcoplasmic reticulum membrane in relation to the excessive production of reactive oxygen species (ROS). Indeed, an increase in both resting levels and exercise-induced production of ROS is often found in ME/CFS patients [8–11, 14–16]. In these studies, thiobarbituric acid reactive substance (TBARS), an element related to overall redox equilibrium and a marker of lipid peroxidation, was used on several occasions to measure oxidative stress. Conversely, oxidation-reduction potential (ORP) per se was not addressed in this context. ORP reflects the capacity of an aqueous medium to either release or accept electrons produced by chemical reactions. ORP therefore measures the overall balance between oxidants and reductants in a system. When the oxidant activity exceeds reductant activity, a high ORP value is measured [17–19].

Because oxidative stress conditions are detected in the blood of ME/CFS patients, we hypothesized that M-wave alterations may not only affect the exercising muscles but also affect resting ones. We therefore investigated the presence of altered muscle excitability in exercising and resting muscles of patients with a clinical diagnosis of ME/CFS. We simultaneously recorded M-waves in the rectus femoris (RF) leg muscle and flexor digitorum longus (FDL) forearm muscle, during and after an incremental cycling exercise. The redox status of the patients was also assessed by measuring resting plasma levels of TBARS and ORP.

## Methods

### Study population

Subjects aged ≥ 18-years referred to our hospital for the diagnosis of chronic fatigue were recruited between April 2018 and June 2019. Inclusion criteria included: at least 6 months of chronic fatigue and at least four symptoms of inability as required for the diagnosis of ME/CFS [1, 2]. Exclusion criteria were: a history of any other pathology confounding the ME/CFS diagnosis, including multiple sclerosis, auto-immune disease (lupus and sicca syndrome), diabetes, congestive heart disease, non-treated hypothyroidism, inflammatory muscle disease, or a significant psychiatric illness (e.g. major depression or psychosis) or pregnancy. Patients with drug abuse during the previous 12 months were also excluded.

The study protocol was approved by the French institutional review boards for human studies (ANSM and CPP Ouest II) and written consent was obtained from all patients to measure their maximal capacity at work.

### Electromyography (EMG) recording and analysis

Bipolar Ag–AgCl surface electrodes (Medtronic, 13 L 20 Skovlunde, Denmark) were used to measure EMG voltage from the RF and FDL muscles on the dominant side of the body, as in previous studies [8–13]. The EMG signal was amplified (Nihon Kohden, Tokyo, Japan; common mode rejection ratio, 90 dB; input impedance, 100 mOhm; gain, 1000–5000) with a frequency band ranging from 10‒10,000 Hz. Compound muscle mass action potentials (M-waves) were evoked by direct muscle stimulation, using a constant-current neurostimulator (Grass, Quincy, MA, USA) delivering supramaximal shocks with 0.1 ms rectangular pulses through an isolation unit. Stimulating skin electrodes were fixed perpendicularly to the muscle axis from either side of the recording electrodes. An oscilloscope (model DSO 400; Gould, Ballainvilliers, France) was used to measure the average M-waves from eight successive potentials and to calculate the peak M-wave amplitude and its duration, and the conduction time, that is the time between the stimulus artefact and the peak.

### Maximal handgrip strength

Maximal handgrip strength was also measured to determine the degree of force failure. Maximal handgrip strength was measured with the wrist in the neutral position with a pronated forearm to hold the handgrip device (model 5401; Takei Scientific Instruments Co Ltd, Niigata-City, Japan), as recommended by de Ponte et al. [20]. Study participants were instructed to perform three maximal handgrips sustained for 3 s. The highest handgrip strength of three contractions, expressed in Newtons (N), was considered the maximum. Each forearm was tested. The reference values were those reported by Steiber [21].

### Blood measurements

Five ml of heparinized blood was taken from each subject to measure the biochemical variables. Potassium concentration was measured at rest and at the end of exercise to evaluate K^+^ outflow in relation to exercise. Two blood markers of oxidative stress (TBARS and ORP) were used. Plasma TBARS concentration was measured according to the method of Uchiyama and Mihara [22]. Plasma ORP was measured using a combined platinum electrode with an Ag/AgCL reference and a potentiostat (ArrowDox; Lazar Research Laboratories, Los Angeles CA, USA). Electrode calibration was performed using 10 different molarities of HCl. Two to three measurements of each plasma sample, separated by a 1-h interval, were performed. Between each measurement, the ORP of pure water was recorded and used to determine the shift with time of the rest value provided by the electrode, which was eventually used to correct the ORP sample values. The results are the average of 2–3 replications with a standard deviation of < 5%.

### Exercise protocol

All patients underwent an exercise session on a cycle ergometer up to their maximal work rate supported for a 1-min period, often limited by muscle fatigue. As in our previous studies [8–11, 13], the protocol consisted of: (i) a 2-min rest period, during which all variables were measured and venous blood samples collected; (ii) a 1-min 20-W work load period used to reach the 1 Hz cycling frequency; (iii) a work period; and (iv) a 10-min recovery period. During the work period, the load was increased by 20 W every 60 s until fatigue obliged the subject to stop the exercise. Peak oxygen uptake (VO_2_max) was measured at the maximal work rate (Wmax) as was the maximal increase in heart rate. M-waves were recorded at 20, 60, 100 W, and at the maximal work rate reached. The ergometer was then unloaded and the subject continued to cycle for a 2-min recovery period to facilitate venous blood return from the legs. During recovery, M-waves were recorded at 2, 5 and 10 min. During the exercise session and the post-exercise recovery period the right forearm remained totally relaxed, the hand being placed on the ergocycle handlebar without grasping.

### Statistical analyses

Power analysis for determination of sample size was founded on an assumption of 95% confidence intervals [95% CI] and 80% power. The Holm-Sidak test was used for both pairwise comparisons and comparisons versus a control situation (rest) to determine the significance of changes in M-wave amplitude and duration. The Student’s t-test was used to assess intergroup differences in M-wave changes between resting values of TBARS and ORP, and maximal changes in M-wave amplitude and duration. Linear regressions between maximal M-wave variations and resting levels of TBARS and ORP were investigated.

## Results

Seventy-two patients were included in the study. After completion of the whole exercise trial, two groups of patients were distinguished: (i) 39 ME/CFS patients without comorbidities with M-wave alterations (group 1); and (ii) 33 ME/CFS patients who did not present M-wave changes (group 2). In group 2, 15 patients suffered from other diseases (obstructive sleep apnoea (n = 4), ankylosing spondylitis (n = 2), sequelae of poliomyelitis (n = 1), Lyme disease (n = 4), Ehlers Danlos syndrome (n = 2) or a psychiatric disorder (burn out) (n = 2)).

There was no difference in age, sex ratio, exercise power characteristics and maximal handgrip strength between the two groups (Table 1), the later observation suggesting that the patients reached the same level of muscle fatigue. There was also no difference in the onset of fatigue. However, the maximal increase in plasma K^+^ concentration, measured at the end of exercise (Delta K^+^ max), was lower and resting TBARS and ORP levels were higher in group 1 vs. group 2.

**Table 1:** Characteristics of the study population

	Group 1 (N = 39)	Group 2 (N = 33)
Age (years)	43 ± 3	47 ± 2
Weight (kg)	64 ± 2	68 ± 2
Sex ratio (F/M)	28/11	17/16
Onset of fatigue (months)	78 ± 19	69 ± 14
TBARS rest (nmol/ml)	1.36 ± 0.03	1.01 ± 0.05***
ORP (mV)	165 ± 5	127 ± 4***
VO_2_max (mlO_2_/min/kg)	19 ± 1	19 ± 1
VO_2_max (% predicted)	67 ± 3	63 ± 3
Maximal work rate (W)	124 ± 5	118 ± 7
Maximal work rate (% predicted)	90 ± 3	83 ± 3
HR max (bpm)	151 ± 3	148 ± 3
Delta K^+^ max (mmol/l)	0.42 ± 0.07	0.65 ± 0.07**
Handgrip strength (N	278 ± 22	315 ± 33
Handgrip strength (% predicted)	62 ± 7	71 ± 7

Group 1 had marked changes in M-wave amplitude and duration whereas no M-wave changes were measured in group 2. All values shown are mean ± standard deviation

*TBARS* thiobarbituric acid reactive substance, *ORP* oxidation reduction potential, *VO*_*2*_ oxygen uptake, *HR* heart rate

Maximal increase in plasma potassium (K^+^) concentration was measured at the end of exercise. Asterisks denote significant intergroup difference (**p < 0.01; ***p < 0.001)

**Fig. 1:**
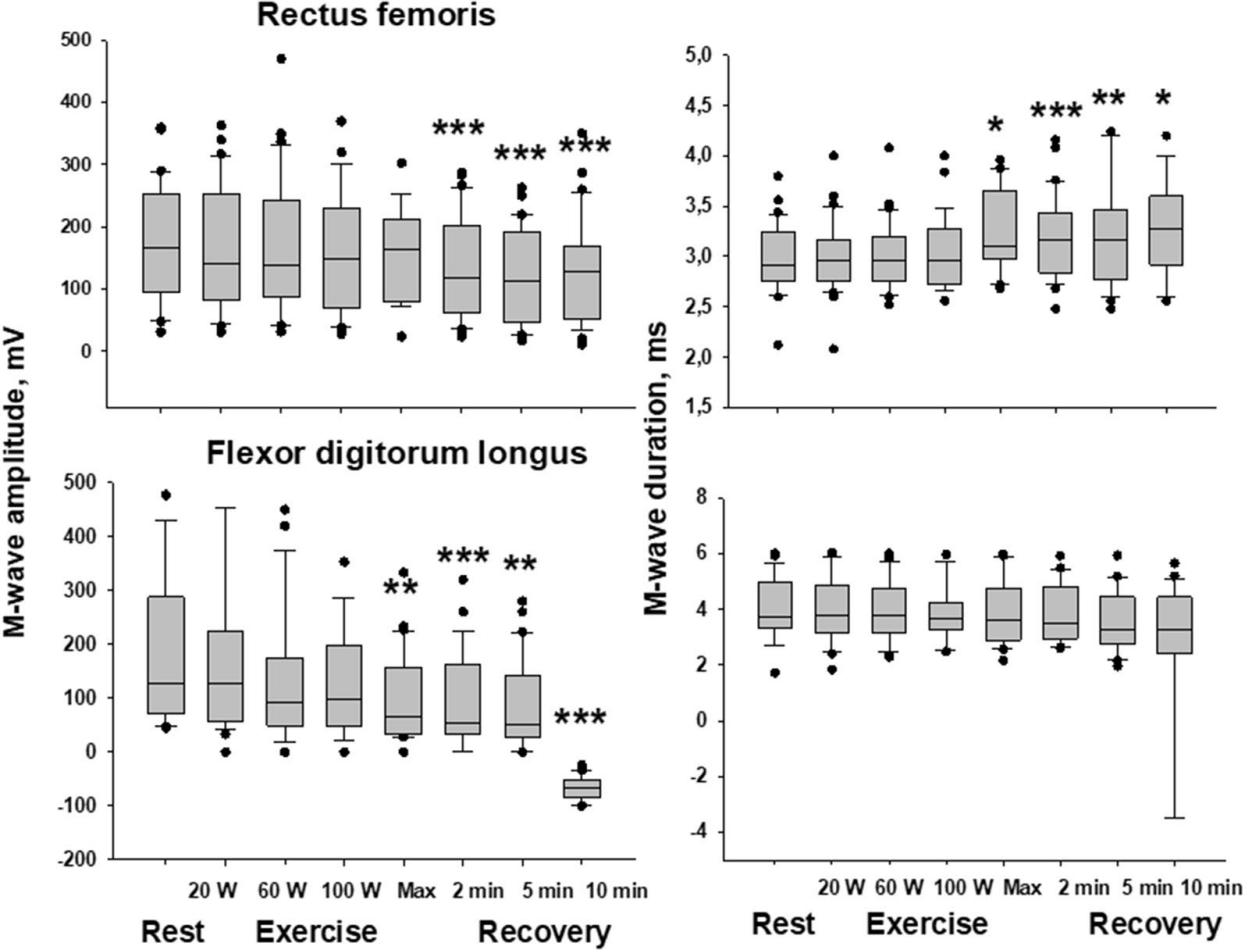
Time course of changes in M-wave amplitude and duration during exercise (rectus femoris) and at rest (flexor digitorum longus) in group 1 patients. Median values are given at rest, at the four steps of exercise, and at the 2nd, 5th, and 10th min of post-exercise recovery. Asterisks (*p < 0.05; **p < 0.01; ***p < 0.001) indicate significant decreases in M-wave amplitude and increased duration

**Fig. 2:**
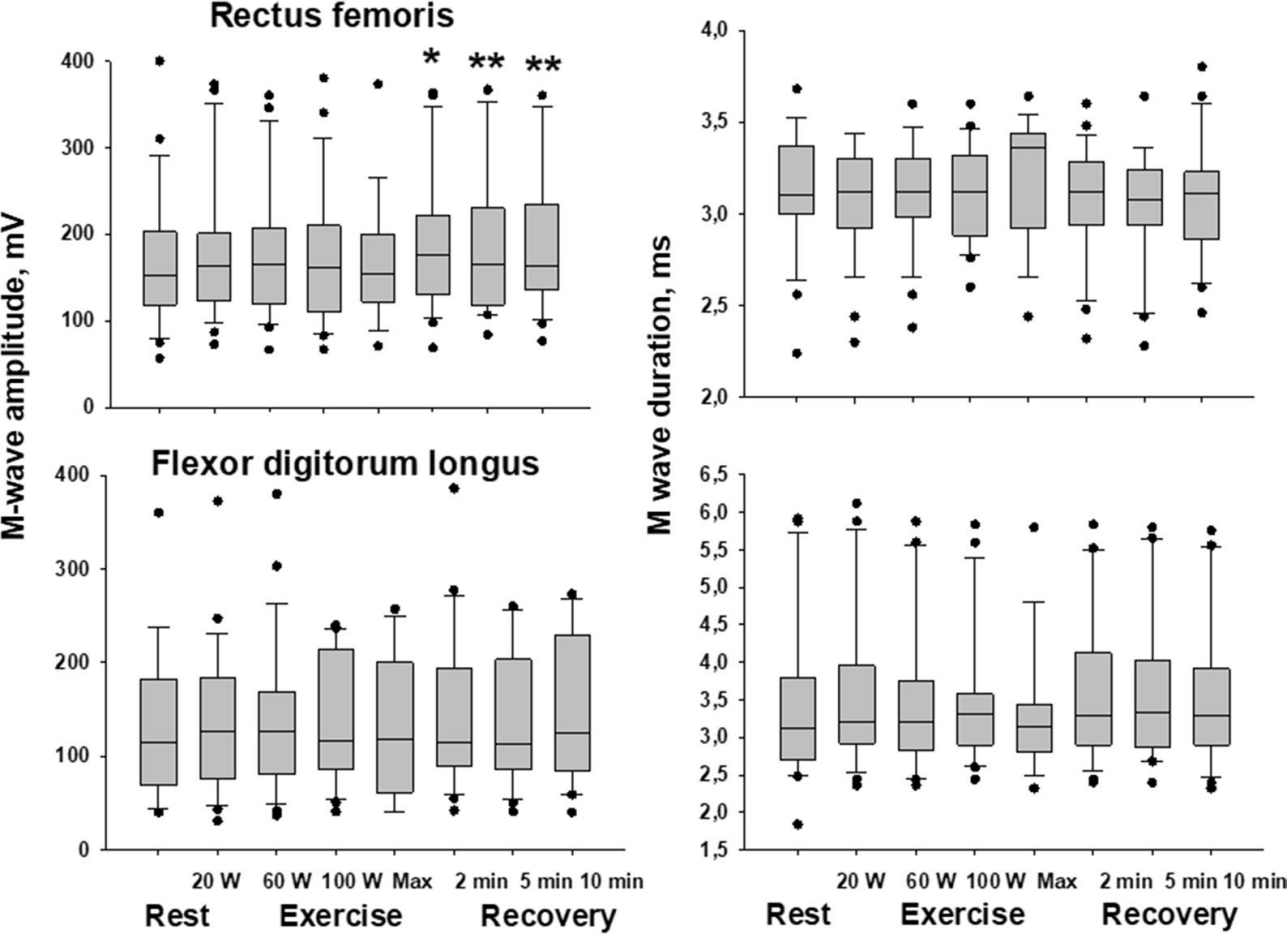
Time course of changes in M-wave amplitude and duration during exercise (rectus femoris) and at rest (flexor digitorum longus) in group 2 patients. Median values are given at rest, at the four steps of exercise, and at the 2nd, 5th, and 10th min of post-exercise recovery. Asterisks (*p < 0.05; **p < 0.01) indicate significant increases in M-wave amplitude

Maximal M-wave variations measured in the RF and FDL muscles are shown in Table 2. In group 1, M-wave alterations were present in both the exercising (RF) and resting (FDL) muscles. More specifically, the M-wave amplitude and length and the conduction time were affected in RF whereas only the M-wave amplitude varied in FDL. The kinetics of the M-wave changes in group 1 are shown in Fig. 1: compared to resting values, significant changes in M-wave amplitude (F value > 12) occurred close to the end of exercise and often progressed until the end of the recovery period. A significant increase in M-wave duration (F = 4.42) only occurred in RF. In group 2, the M-wave amplitude tended to increase in RF (F = 10.24) with no change in duration or conduction time. No M-wave change occurred in FDL (Table 2 and Fig. 2).

**Table 2:** M-wave characteristics

	Group 1 (N = 39)	Group 2 (N = 33)
Rectus femoris		
Delta M wave amplitude (%)	− 41 ± 4^†^	+ 14 ± 3***
Delta M wave duration (%)	+ 18 ± 3^#^	+ 2 ± 3***
Delta M wave latency (ms)	+ 2.65 ± 0.17	+ 0.07 ± 0.04***
Flexor digitorum longus		
Delta M wave amplitude (%)	− 65 ± 4^†^	+ 22 ± 5***^,#^
Delta M wave duration (%)	+ ± 4	+ 8 ± 2
Delta M wave latency (ms)	+ 0.26 ± 0.07	+ 0.04 ± 0.05**

Maximal variations in M-wave amplitude and duration expressed as a percentage of resting values. Latencies measured at rest and at the end of the post-exercise recovery period. Symbols indicate that the changes in each group differ significantly from control values (i.e. rest data) (^#^p < 0.01; ^†^p < 0.001). Asterisks denote significant intergroup differences (***p < 0.001). Post-exercise variations in M-wave latencies are not significant

**Fig. 3:**
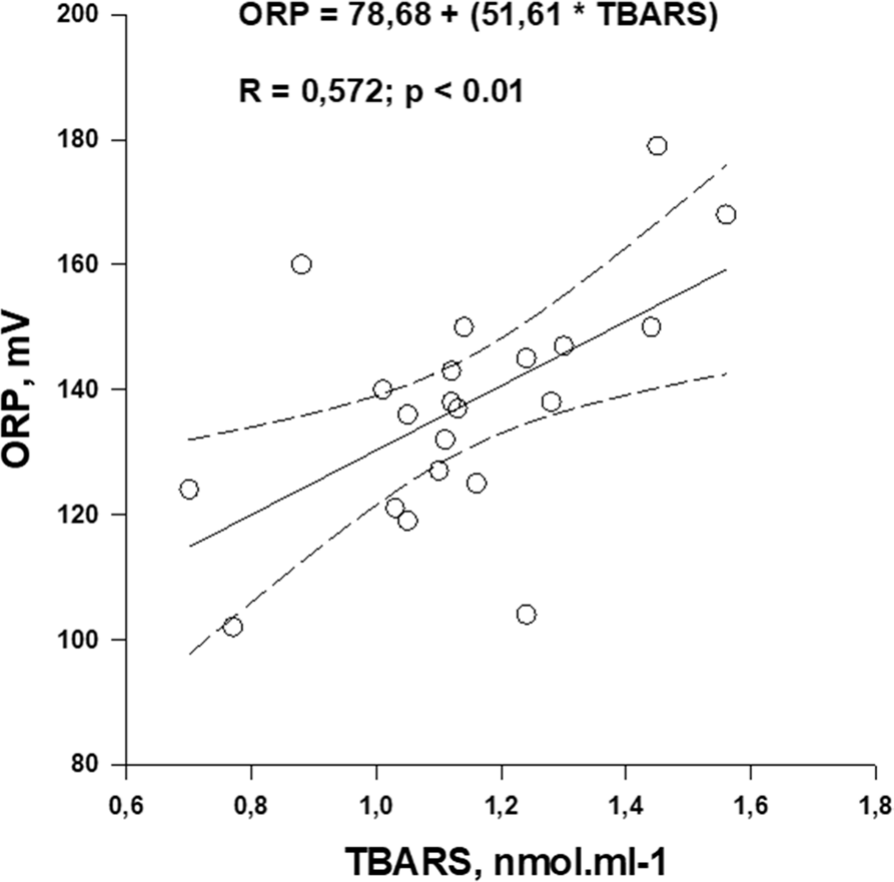
Regression with 95% CIs obtained between resting TBARS and ORP values. The regression equation and significance against zero of the R coefficient value is indicated

**Fig. 4:**
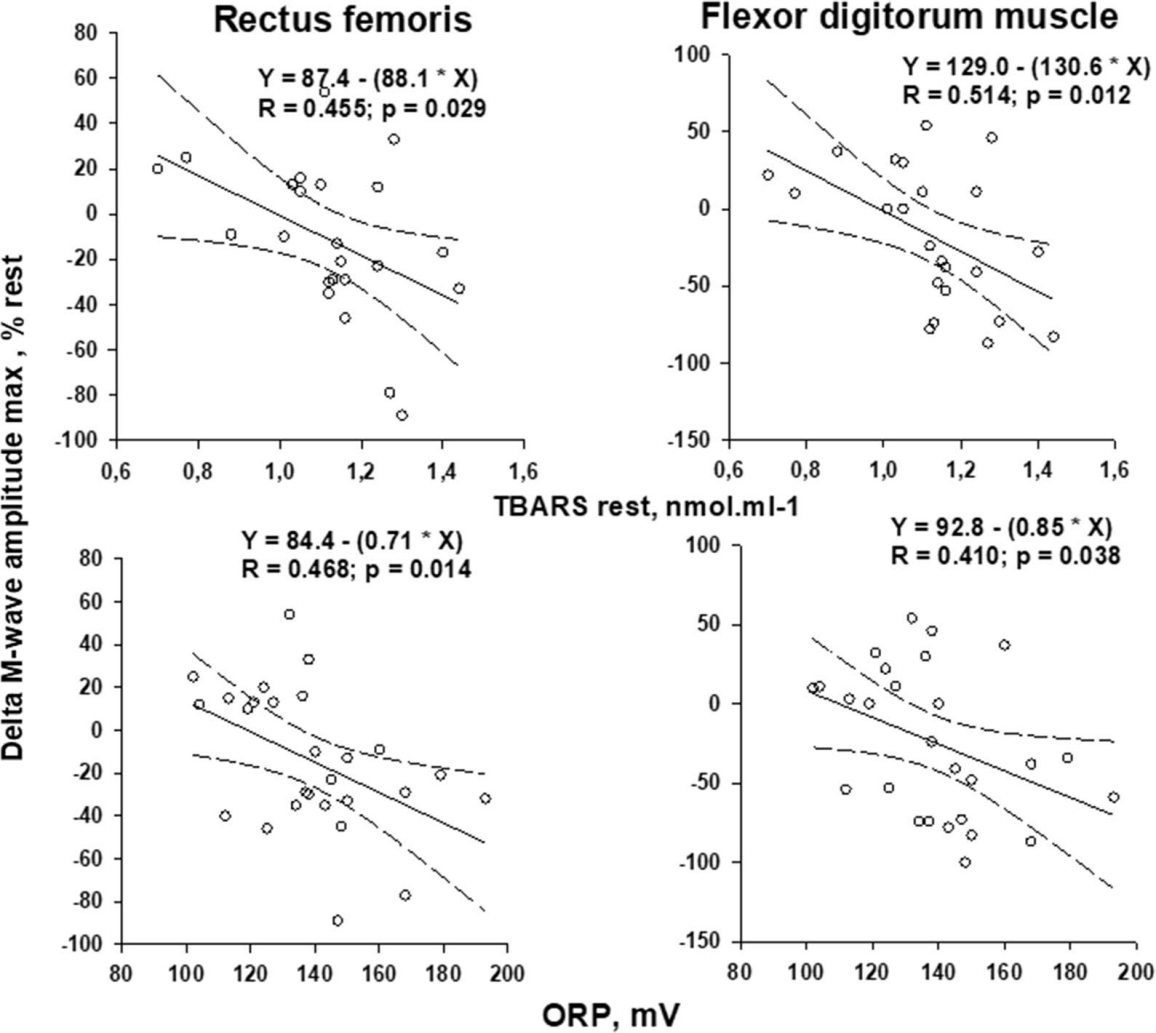
Maximal variations in M-wave amplitude expressed as a percentage of resting values plotted against resting levels of TBARS and ORP. Linear regressions with 95% CIs are shown as well as the regression equations and the significance against zero of the R coefficient value

Regarding plasma markers, a significant positive correlation was found between TBARS and ORP values (Fig. 3). When plotting all values measured in both groups of patients (i.e. maximal variations in M-wave amplitude vs. resting levels of TBARS or ORP), negative linear regressions were found (Fig. 4). No significant correlations were found between M-wave changes in the two muscle groups and K^+^ outflow magnitude.

## Discussion

This study distinguished two groups of patients with ME/CFS. In group 1, M-wave alterations were found during and after exercise in both the exercising leg muscle and the resting forearm muscle. In the exercising leg muscle, a decreased in amplitude and longer duration of M-waves were measured whereas in the resting forearm muscle only an alteration in M-wave amplitude was observed. By referring to the redox data presented here, our study shows that high ORP and TBARS values were associated with disorders in both exercising and resting muscles. Finally, exercise-induced K^+^ outflow was reduced and M-wave alterations were proportional to the magnitude of oxidative stress. In group 2, with no M-wave alterations, low baseline levels of TBARS and ORP were measured. It should be noted however that half the subjects in this group suffered from comorbidities which could contribute to body fatigue.

There are several important strengths of this study. First, we used a population with homogeneous criteria for ME/CFS. Second, all patients completed a blinded exercise protocol.

The main limitation to this study is its monocentric nature. Thus, we could not exclude potential referral bias.

The present observations confirm the existence of redox disorders in many ME/CFS patients [3–11, 14–16]. These data also show that abnormal redox status is generally present in ME/CFS patients with abnormal muscle membrane excitability, in contrast to patients with no muscle abnormalities. We therefore conclude that high ORP values can be used to distinguish these two groups of patients. A recent study has already reported that plasma ORP changes give a rapid, accurate and user-friendly measurement of increased oxidative stress [23]. We confirm this idea and conclude that ORP measurement is cheaper, quicker to perform and provides information on the overall imbalance between oxidants and anti-oxidants whereas TBARS measurements need complex biochemical processing and only explore a single element of the overall redox equilibrium.

The present study also shows that the decreased M-wave amplitude in both exercising and resting muscles was proportional to the magnitude of oxidant/anti-oxidant imbalance in plasma, which supports the primary hypothesis of Fulle et al. [14]. This also supports the hypothesis that M-wave alterations in non-exercising muscles result from a systemic disorder of muscle membrane excitability due to dysregulation of the oxidant/anti-oxidant balance in blood. Increased ROS production following exercise inhibits Na^+^–K^+^ pump activity [7], reducing the K^+^ outflow and muscle membrane excitability [24]. Previous studies have shown that increased plasma oxidant level is associated with reduced K^+^ outflow from exercising muscles in healthy subjects [25] and ME/CFS patients [10]. In the present study, it should be noted that K^+^ outflow measured at the end of exercise was higher in group 2 vs. group 1. This indicates that the dysregulation of oxidant/anti-oxidant status in plasma alters the ionic flux through the muscle membrane, which may partly promote altered muscle membrane excitability.

The existence of a disorder of muscle excitability after exercise could partly explain the occurrence of PEM reported by many ME/CFS patients. However in our series of patients PEM was present in both groups. Pietrangelo et al. [26] hypothesized that patients with ME/CFS are subjected to problems of muscle aging (sarcopenia) and that intergroup differences in biological data (M-wave, redox status, K^+^ outflow) may have resulted from differences in gene expression. Gene expression subtypes have been reported in ME/CFS patients [27] and the authors isolated 40 abnormal gene pathways. However, the intergroup differences between oxidative abnormalities could not result from age and/or sex differences as reported in healthy subjects by Fano et al. [28] because in our study these parameters were similar in the two groups. Furthermore, the comorbidities present in group 2 patients cannot explain the absence of marked oxidative damage. Indeed, high levels of oxidative stress are often measured in patients with ankylosing spondylitis [29] or Lyme disease [30], these comorbidities being present in six of our patients. Additionally, fatigue that leads to physical deterioration is another important symptom that is observed in 90% of patients with post-polio syndrome, probably resulting from spine frontal horn motor neuron damage during acute poliovirus infection [31]. However this comorbidity was only reported in one of our patients. We therefore have no satisfactory explanation for the intergroup differences observed here.

## Conclusion

Future studies in different patient populations may help to further refine the biological criteria of ME/CFS and in particular address the reason(s) for the presence or absence of peripheral muscle fatigue in patients. Our data also highlight the heterogeneity of some biomarker measurements in patients with a diagnosis of ME/CFS. Finally, our data support the use of ORP to address the redox stress in a ME/CFS context with diagnostic relevance.

## Data Availability

The datasets used and/or analyzed during the current study are available from the corresponding author on reasonable request.

## Abbreviations

CFS: Chronic fatigue syndrome
EMG: Electromyography
FDL: Flexor digitorum longus
K^+^: Potassium
ME: Myalgic encephalomyelitis
M-wave: Evoked muscle action potential
ORP: Oxidation–reduction potential
PEM: Post-exertional malaise
RF: Rectus femoris
ROS: Reactive oxygen species
TBARS: Thiobarbituric acid reactive substance
VO_2_max: Maximal oxygen uptake
Wmax: Maximal work rate
